# Learning from experience: does providing end-of-life care support for relatives boost personal end-of-life health literacy?

**DOI:** 10.1186/s12904-025-01645-1

**Published:** 2025-01-09

**Authors:** Clément Meier, Maud Wieczorek, Sarah Vilpert, Carmen Borrat-Besson, Ralf J. Jox, Jürgen Maurer

**Affiliations:** 1 Faculty of Biology and Medicine (FBM) & The faculty of Business and Economics (HEC), Swiss Centre of Expertise in the Social Sciences (FORS), University of Lausanne, Lausanne, Switzerland; 2 Faculty of Business and Economics (HEC), University of Lausanne, Lausanne, Switzerland; 3 Faculty of Business and Economics (HEC), Swiss Centre of Expertise in the Social Sciences (FORS), University of Lausanne, Lausanne, Switzerland; 4 Swiss Centre of Expertise in the Social Sciences (FORS), University of Lausanne, Lausanne, Switzerland; 5 Palliative and Supportive Care Service, Chair in Geriatric Palliative Care, Institute of Humanities in Medicine, Lausanne University Hospital and University of Lausanne, Lausanne, Switzerland

**Keywords:** End of life, Health literacy, Caregivers, Experiences, Family care

## Abstract

**Background:**

Despite the critical role of health literacy in utilizing palliative care and engaging in advance care planning, limited research exists on the determinants of end-of-life health literacy. This study investigates the association between individuals’ experiences with end-of-life care support to relatives and their end-of-life health literacy among a population-based sample of adults aged 58 and older.

**Method:**

We used data from 1,548 respondents in Switzerland to Wave 8 (2019/2020) of the Survey on Health, Ageing, and Retirement in Europe. Their ability to understand medical jargon, find information, communicate, and make decisions about end-of-life care options was measured with the validated Subjective End-of-Life Health Literacy Scale. Experiences with end-of-life care support include having made medical decisions as healthcare proxy, accompanied, or cared for relatives at the end of life. Associations were estimated using ordinary least squares regressions, controlling for socio-demographic, health, and regional characteristics.

**Results:**

Respondents who experienced being a healthcare proxy (*p* < 0.001), who accompanied (*p* < 0.001), or who cared for a relative at the end of life (*p* < 0.001) tended to have higher levels of end-of-life health literacy. These results remained significant when the three variables were simultaneously included in the multivariable model (*p* < 0.001, *p* < 0.001 and *p* < 0.05).

**Conclusions:**

Our findings suggest that providing end-of-life care support to relatives is associated with higher end-of-life health literacy. Thus, as caregivers gain experience caring for others, targeted interventions could leverage their skills and encourage them to think of engaging in end-of-life planning for themselves.

**Supplementary Information:**

The online version contains supplementary material available at 10.1186/s12904-025-01645-1.

## Background

Demographic aging and the increasing medicalization of end-of-life care over recent decades have significantly transformed the societal and healthcare context surrounding end-of-life issues [1]. Most deaths in European countries including Switzerland occur at older age due to chronic conditions like cardiovascular diseases and cancer [2], with neurodegenerative conditions and frailty shaping prolonged end-of-life trajectories [3, 4], often involving one or multiple hospital admissions in the final phase of life [5, 6]. This evolving context underscores the importance of acknowledging and respecting individual healthcare preferences, particularly in preference-sensitive decisions, such as whether to pursue aggressive medical treatments or prioritize palliative care, which often involve complex trade-offs between quality and length of life [7, 8]. As a result, in recent years, there has been a significant rise in the necessity to make critical healthcare decisions towards the end of life [9]. End-of-life decisions refer to the choices individuals make regarding their healthcare and treatments as they approach the final stages of life [10]. These may involve discussions with healthcare professionals about advance care planning [11], decisions on whether to receive aggressive medical interventions, or opting for palliative or hospice care, and can vary based on the individual’s preferences and medical condition [12]. However, end-of-life decisions present their own set of challenges, as they are often made in emotionally charged situations involving life-and-death questions, offer multiple options with prognostic and other uncertainties, and involve complex trade-offs between the length and quality of life, usually being made without preparation and often on behalf of patients with impaired decision-making capacity [13]. It is, therefore, essential to provide individuals with the necessary competencies to make informed decisions about challenging end-of-life situations they may not be familiar with and in which they might lack appropriate knowledge [14].

To effectively support individuals in making end-of-life healthcare decisions, ensuring they possess adequate skills is crucial, guaranteeing they are well-informed, empowered, and supported throughout their decision-making processes [15]. Health literacy, defined by the US Centers for Disease Control and Prevention as “the degree to which individuals have the ability to find, understand, and use information and services to inform health-related decisions and actions for themselves and others” [16, 17], plays a vital role in enhancing patient autonomy, improving satisfaction, and achieving better healthcare outcomes [18, 19]. While health literacy plays a crucial role, specialized measures are needed to address the unique challenges of end-of-life decision-making and to accurately assess the distinct competencies required for navigating complex end-of-life healthcare decisions [20]. In the context of end-of-life care, several specialized forms of literacy have emerged to address the unique challenges individuals face at the end of life. For instance, Death Literacy focuses among other aspects on individuals’ knowledge, skills, and understanding of the death system, enabling them to make informed decisions about death care options [21], while Grief Literacy encompasses knowledge, skills, and values to enable compassionate action and support for those grieving [22]. However, when it comes to the specific challenges of making healthcare decisions at the end of life, End-of-life health literacy has recently been developed to assess individuals’ perceived abilities to navigate the complexities of end-of-life planning and decision-making [23]. End-of-life health literacy encompasses more than just understanding medical terminology; it involves making informed decisions, communicating wishes, and knowing where to find help [23]. Previous studies have indicated that inadequate health literacy related to end-of-life is linked to communication challenges between patients and healthcare providers regarding end-of-life care [24], confusion regarding treatment options at the end of life [25], a higher likelihood of receiving aggressive care during end-of-life [26], reduced participation in advance care planning [27, 28], and decreased likelihood of possessing an advance directive [29]. In addition, while individuals’ end-of-life health literacy is critically important, existing studies suggest that patients’ comprehension of end-of-life care options is often suboptimal [30, 31], indicating a possible lack of essential skills to effectively manage end-of-life medical situations. This highlights the need to explore the determinants of end-of-life health literacy that can enhance individuals’ ability to navigate these critical healthcare decisions.

One key area that need further attention is the role of caregiving experiences as it could potentially significantly enhance end-of-life health literacy by providing individuals with firsthand exposure to complex decision-making and care management. During the final stages of life, individuals often encounter unique and complex challenges that can impact their physical health, and as their medical needs increase and decision-making becomes more complex, informal caregivers become crucial as they guide patients through their illness journey, providing essential care and emotional support [32]. Caregivers support individuals living with chronic illnesses or experiencing cognitive or physical challenges by providing them with various types of support: practical, emotional, physical, and social [33]. Additionally, caregivers often assume the challenging role of making end-of-life decisions [34], these experiences can help them better understand end-of-life care options and engage more proactively in advance care planning [35].

However, despite the vital role caregiving plays in supporting patients, there remains a gap in research examining how caregiving experiences may specifically enhance individuals’ end-of-life health literacy [36]. This study, thus, aims to investigate whether personal experiences in providing end-of-life care support to relatives is associated with end-of-life health literacy among a population-based sample of adults aged 58 and older. Understanding this relationship could inform targeted interventions, empowering caregivers with critical skills and knowledge, ultimately leading to more informed, proactive participation in end-of-life care planning for themselves and their relatives.

## Methods

### Study design and participants

The study analyzed responses from participants who completed a Switzerland-specific questionnaire distributed as part of the Wave 8 of the Survey on Health, Ageing, and Retirement in Europe (SHARE) collected from October 2019 to early March 2020 [37, 38]. SHARE is a comprehensive longitudinal research initiative that has been collecting in-depth data on health, socio-economic status, and social and family networks from targeted respondents and their partners aged 50 and over across 27 European countries and Israel. In Switzerland, 2,005 targeted respondents and their partners participated in the Wave 8 in-person interviews, with a majority of 1,891 (94.3%) also completing the additional national paper-and-pencil questionnaire. Since the Swiss population sample of SHARE participants aged 50 and above has not been refreshed since 2011, partners from targeted respondents in the 50–57 age range were excluded from the present study to avoid potential sampling bias, as this age group may not accurately represent the current population. The study thus only includes individuals aged 58 years and above. From the 1,891 respondents who completed the national paper-and-pencil questionnaire, 28 respondents younger than 58 years old, and an additional 315 respondents with incomplete answers to relevant variables were excluded from the analysis. Consequently, the final sample size consisted of 1,548 respondents for the present study.

### Outcome variable

#### Subjective End-of-life Health Literacy Scale (S-EOL-HLS)

The scale evaluates participants’ perceived end-of-life health literacy abilities, focusing specifically on decision-making skills [23]. It includes 18 questions, detailed in Appendix S1, organized into three dimensions: (1) Functional end-of-life health literacy, evaluating participants’ self-assessment of their understanding of medical terminology related to end-of-life care; (2) Interactive end-of-life health literacy, which evaluates the participants’ confidence in setting treatment objectives, sourcing information about end-of-life care, and discussing end-of-life issues; and (3) Critical end-of-life health literacy, where participants reflect on their ability to make informed choices regarding medical treatments. Each question on the scale is rated using a 4-point Likert scale, where options range from “very easy” to “very difficult.” These responses are subsequently recoded in a binary variable with “very difficult” and “fairly difficult” assigned as a score of “0,” while “very easy” and “fairly easy” scored as “1”. This coding results in a score ranging from 0 to 18 with higher scores reflecting better end-of-life health literacy. As per Pelikan et al.‘s methodology (2019) [39], any missing responses are counted as “0,” with a final end-of-life health literacy score being calculated for those with no more than two missing responses. Out of the total respondents, 116 (6.8%) had over two non-responses on the 18 questions. The final score is then normalized by dividing it by the standard deviation (4.6), yielding a score between 0 and 3.9.

### Exposure

#### Experiences providing end-of-life care support

The national paper-and-pencil questionnaire administered in Wave 8 included three questions on different types of end-of-life care support participants may have provided. The first question asked if participants had ever made medical decisions on behalf of a person at the end of life who was close to them and who was no longer able to decide for him or herself (Yes/No). The second question asked whether participants had ever accompanied (through physical presence, visiting, or providing moral support) a relative or close friend at the end of life (Yes/No). And finally, the last question inquired if they had ever cared (personal care, giving medicine, feeding) for a relative or close friend at the end of life (Yes/No).

### Covariates

The statistical analysis considered the following key demographic and socio-economic variables. These included sex (categorized as male or female), age groups (split into three brackets: 58–64 years, 65–74 years, and 75 years or older), and education levels (classified as low, corresponding to International Standard Classification of Education (ISCED) levels 0-1-2; middle, equivalent to ISCED levels 3–4; and high, aligning with ISCED levels 5–6) [40]. Additionally, partnership status (whether the individual has a partner or not), the language region in Switzerland (German, French, or Italian), subjective financial status (measured by the ease of meeting financial needs: easily, fairly easily, or with difficulty), type of living area (urban or rural), and self-assessed health status (categorized as poor/fair, good, or very good/excellent health) were also considered.

### Statistical analysis

The demographic details of the study’s participants were presented through the use of numerical counts and percentage distribution. Bar charts were used to display the bivariate relationships between the three distinct experiences of end-of-life care support and the mean standardized scores of end-of-life health literacy. In addition, the partial associations between the three types of experiences of end-of-life care support and the standardized end-of-life health literacy score were assessed using ordinary least squares regression models controlling for sex, age, education levels, partnership status, Switzerland’s linguistic regions, subjective financial difficulties, living area, and self-rated health. The analysis first explored the individual associations between each caregiving experience and the standardized end-of-life health literacy score using separate ordinary least squares regressions. It then included all three exposure variables simultaneously in one final model. Furthermore, error terms were grouped by household to consider possible unseen interdependencies among the targeted respondents and their partners. The analysis utilized STATA/SE 17.0 software (STATA Corporation, College Station, TX) for all calculations. Two-sided p-values < 0.05 were considered statistically significant with outcomes presented as average marginal effects (AME) and the associated standard errors (SE).

## Results

Table 1 outlines the demographic characteristics of the study participants. In total, 52.9% of participants were female. Regarding their age, 24.7% fell within the 58–64 years range, 42.3% were aged between 65 and 74 years, and the remaining 33.0% were 75 years or older. When examining educational attainment, the majority, 63.2%, reported having a middle level of education, while 19.6% had a high level, and 17.2% had a low level of education. With regards to partnership status, 75.3%, indicated that they had a partner, whereas 24.7% did not. In terms of financial ease, a majority of 55.3% reported being able to make ends meet easily, 31.8% fairly easily, and 12.9% with difficulty. The linguistic distribution of the sample was predominantly German-speaking (71.3%), followed by French (25.0%) and Italian speakers (3.7%). Geographically, 54.8% resided in rural areas, compared to 45.2% in urban areas. Regarding self-rated health, 18.1% of the respondents considered their health to be poor or fair, while 42.3% rated it as good, and 39.5% as very good or excellent. Concerning experiences with end-of-life care, 26.2% had made medical decisions for someone at the end of life, 65.7% had accompanied a person at the end of life, and 30.4% had cared for someone at the end of their life.

**Table 1:** Characteristics of the study population, adults aged 58+, SHARE Switzerland, 2019/2020, *n* = 1,548

	*n*	%
**Gender**		
Male	729	47.1
Female	819	52.9
**Age groups**		
58–64 years	383	24.7
65–74 years	655	42.3
75 + years	510	33.0
**Education**		
Low	267	17.2
Middle	978	63.2
High	303	19.6
**Partnership status**		
Has a partner	1,165	75.3
No partner	383	24.7
**Make ends meet**		
Easily	856	55.3
Fairly easily	492	31.8
With difficulty	200	12.9
**Language**		
German	1,104	71.3
French	387	25.0
Italian	57	3.7
**Living area**		
Urban	700	45.2
Rural	848	54.8
**Self-rated health**		
Poor/fair health	281	18.1
Good health	655	42.3
Very good/excellent health	612	39.5
**Made medical decision**		
No	1,143	73.8
Yes	405	26.2
**Accompanied someone**		
No	531	34.3
Yes	1,017	65.7
**Cared for someone**		
No	1,077	69.6
Yes	471	30.4
**End-of-life health literacy score standardized**	mean: 2.9 min: 0	std. dev: 1 max: 3.9

Note, number of observations for the whole sample

**Fig. 1:**
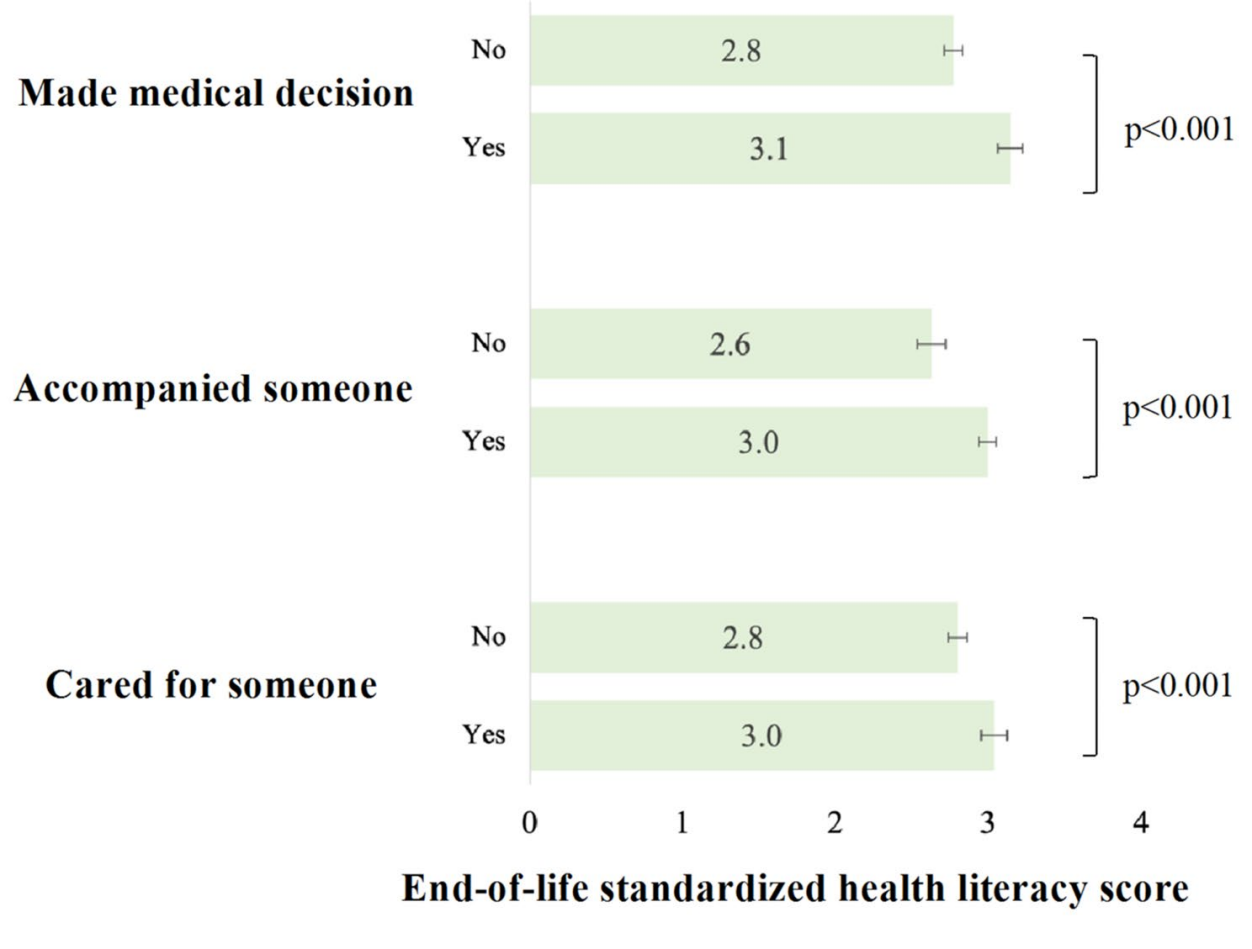
Average standarized scores of end-of-life health literacy by types of end-of-life care support, adults aged 58+, SHARE switzerland, 2019/2020, n = 1548

The bivariate associations presented in Fig. 1 describe the relationship between the three types of end-of-life care support and the standardized end-of-life health literacy score. The results show that respondents who made medical decisions, accompanied someone at the end of life, or cared for someone at the end of life had systematically higher standardized end-of-life health literacy scores, compared to respondents who did not engage in these caregiving activities (*p* < 0.001).

Table 2 illustrates the partial associations between the three types of end-of-life care support and the standardized end-of-life health literacy scores, adjusted for several covariates including sex, age, education levels, partnership status, subjective financial situation, linguistic region, living area, and self-rated health. The results show that individuals who made medical decisions for someone at the end of their life (AME: 0.26, *p* < 0.001), those who accompanied a relative or friend at the end of life (AME: 0.29, *p* < 0.001), and those who provided care to someone at the end of life (AME: 0.23, *p* < 0.001) had higher end-of-life health literacy scores compared to individuals who did not provide any of the three types of end-of-life care support. The results remained significant when the three variables were simultaneously included in the multivariable model (AME: 0.18, *p* < 0.001; AME: 0.21, *p* < 0.001; and AME: 0.12, *p* < 0.05, respectively).

**Table 2:** Partial associations between standardized scores of end-of-life health literacy on the three types of end-of-life care support, adults aged 58+, SHARE Switzerland, 2019/2020, *n* = 1,548

	Model 1 End-of-life health literacy	Model 2 End-of-life health literacy	Model 3 End-of-life health literacy	Model 4 End-of-life health literacy
Made medical decision	0.26^***^ (0.05)			0.18^***^ (0.05)
Accompanied someone		0.29^***^ (0.05)		0.21^***^ (0.06)
Cared for someone			0.23^***^ (0.05)	0.12^*^ (0.06)
Observations	1,548	1,548	1,548	1,548

The table shows average marginal effects and standard errors in parentheses. Statistical significance: * *p* < 0.05, ** *p* < 0.01, *** *p* < 0.001. Models 1, 2 and 4 show the results from ordinary least squares regressions of the standardized scores of end-of-life health literacy on each of the three types of end-of-life care support and the covariates. Model 4 shows the results from ordinary least squares regressions of the standardized scores of end-of-life health literacy on the three types of end-of-life care support and the covariates. The covariates include sex, age, education levels, partnership status, subjective financial situation, linguistic region, living area and self-rated health

## Discussion

Using a population-based sample of 1,548 adults aged 58 and older in Switzerland, this study investigates the association between individuals’ experiences with end-of-life care support to relatives and end-of-life health literacy. The findings demonstrate a positive association, suggesting that personal caregiving experience is associated with higher end-of-life health literacy. More precisely, individuals who either made medical decisions for someone at the end of their life, accompanied a relative or friend or provided care to someone at the end of life had higher end-of-life health literacy scores compared to those who did not. Understanding the association between personal caregiving and increased end-of-life health literacy suggests the potential for targeted interventions.

### Caregivers’ health literacy

Health literacy is crucial for enhancing autonomy and satisfaction in healthcare, particularly for the aging population at risk of chronic diseases, as it influences their understanding of health challenges, communication with healthcare providers, and capacity to make informed, autonomous medical decisions outcomes [20, 41]. When considering the involvement of caregivers in making healthcare decisions, managing complex medical treatments at home, and engaging in health information exchange, it seems critical to evaluate their health literacy to ensure the best health outcomes for those they care for. Previous studies showed that low health literacy in caregivers was associated with several outcomes: poorer self-management behaviors in care recipients, increased usage of health services by care recipients, and a heightened sense of burden on the caregivers [36]. Having adequate health literacy skills is essential for caregivers, particularly in end-of-life care, yet the process by which they develop these necessary skills in such a complex setting remains uncertain.

### Learning by experiences

Caregivers often seek various types of information, including details about the patient’s illness, its progression and prognosis, available treatment options, and potential symptoms and side effects [42]. To obtain this information, caregivers engage in experiential learning, acquiring skills through various methods: experimenting and learning from mistakes, actively seeking necessary information and advice, applying knowledge and skills from previous experiences, and reflecting on their current experiences [43]. In a previous study, three primary areas of learning were identified: understanding the illness and its progression, mastering the skills required for caregiving, and learning how to seek and access necessary assistance [44]. These learning experiences align with the ones measured in our study, for instance, making medical decisions often requires caregivers to interact with healthcare providers and understand complex medical information, which can enhance their ability to navigate the healthcare system and communicate effectively. Managing treatments at home may involve hands-on experience with medical procedures, medications, and symptom management, which fosters a deeper understanding of care processes. Additionally, providing emotional support helps caregivers develop the skills needed to facilitate difficult conversations about care preferences, prognosis, and end-of-life wishes. These diverse experiences allow caregivers to acquire and strengthen the competencies needed for informed decision-making and engagement in end-of-life planning, which may explain the observed associations with increased end-of-life health literacy. However, even though the learning process of caregivers remains self-driven by discovery and experiences, they typically prefer and appreciate a learning approach that is supported or guided, involving instructions or demonstrations by healthcare professionals [43]. It underscores the importance of supporting caregivers with structured guidance and resources to enhance their ability to navigate the complexities of end-of-life care effectively, ultimately enriching their caregiving experience and the quality of care they provide.

### Practical implications and future research

The findings of the present study support the hypothesis that caregivers, through their experience in caring for others, may be more likely to have higher end-of-life health literacy. Similarly, a recent study.

shows that individuals who had previous involvement in making medical decisions for a relative were significantly more likely to engage in their own advance care planning conversations with family members [35]. This relationship was mediated by their knowledge of their relative’s end-of-life treatment preferences [35]. This suggests that caregiving experiences can facilitate better understanding and communication about end-of-life issues, reinforcing the idea that these experiences may enhance end-of-life health literacy. Caregivers who have navigated these complex decisions may be better equipped to anticipate and engage in similar discussions for themselves, which supports the hypothesis that caregiving experiences are linked to higher levels of end-of-life health literacy. Therefore, targeted interventions could utilize insights from caregivers’ experiences to enhance communication strategies specifically aimed at both family caregivers and healthcare professionals.

For family caregivers, these strategies could focus on providing them with the tools and knowledge to communicate more effectively with healthcare providers, express their needs and preferences, and navigate end-of-life care decisions. One intervention could be, for instance, to invite caregivers to participate in a training. As an example of effective interventions, the Scottish Partnership for Palliative Care offers a free course called End of Life Aid Skills for Everyone (EASE) [45]. This course helps members of the public support those dealing with death, dying, and bereavement by building confidence and addressing the emotional and practical challenges of end-of-life care. Available in both face-to-face and online formats, EASE promotes learning through group discussions, short films, and peer support activities. Similarly, the “Last Aid” course is an international public education initiative, often described as “first aid for end-of-life” [46]. Developed in Germany and now offered in 23 countries equips participants with essential knowledge and skills to support loved ones through the dying process. It covers topics such as palliative care, pain management, emotional support, and discussions about death, aiming to reduce fear and increase confidence in providing end-of-life care. Both courses demonstrate how public education can enhance end-of-life care support by improving communication and practical caregiving skills.

For healthcare professionals, interventions could center on improving their ability to engage caregivers in meaningful, compassionate conversations about end-of-life options and planning. For instance, The DöBra cards, a tool designed to facilitate conversations about end-of-life care, offer healthcare professionals a practical method to initiate and guide discussions with older adults. By using these cards, professionals can “break the ice” on sensitive topics, helping individuals reflect on their values and preferences for end-of-life care [47]. By fostering better communication, these interventions could spark broader interest in end-of-life issues among the general population, encouraging individuals to proactively seek information and prepare for potential caregiving roles. Recognizing that many individuals may eventually become caregivers themselves, facing complex decisions on behalf of their relatives, these interventions could motivate them to enhance their understanding of end-of-life care early on. Moreover, as caregivers accumulate experience in caring for others, new interventions could capitalize on their skills and encourage them to consider engaging in their own end-of-life planning. Additionally, future research should monitor changes in end-of-life health literacy over time through longitudinal studies and investigate whether experiences in providing end-of-life care to relatives serve as a catalyst for developing end-of-life health literacy skills.

### Limitations

Our study recognizes several limitations. Firstly, subjective measures like the S-EOL-HLS, while widely used, are prone to reporting biases as participants’ familiarity with end-of-life issues might lead to underestimating or exaggerating their skills. Secondly, the S-EOL-HLS covers only some aspects of end-of-life health literacy skills, suggesting there might be more to explore to fully understand the scope and applicability of the data. Also, while SHARE aims to accurately represent Switzerland’s older population, issues such as attrition in longitudinal studies and item nonresponse could affect our results. Nevertheless, the robust response rate in the Swiss questionnaire and the absence of any critical tendency when regressing on the covariates the participants excluded due to missing values in the variables used in the analysis lend some confidence to our conclusions. Finally, the cross-sectional design of this study restricts our ability to draw causal conclusions, calling for further research. It is also important to acknowledge that individuals with higher end-of-life health literacy may be more likely to engage in end-of-life situations, rather than caregiving experiences necessarily leading to increased end-of-life health literacy. Therefore, while our findings highlight an association, they do not confirm causation. Future longitudinal research is needed to explore whether caregiving experiences actively contribute to the development of end-of-life health literacy or if individuals with higher end-of-life health literacy are more predisposed to take on caregiving roles and engage in end-of-life planning.

## Conclusion

The study involving 1,548 Swiss adults aged 58 and older revealed a significant association between providing end-of-life care to relatives and increased end-of-life health literacy. Specifically, individuals who made medical decisions, accompanied a friend or relative at the end-of-life, or provided care at the end of life demonstrated higher end-of-life health literacy scores. The results open avenues for targeted interventions that could guide interventions to raise awareness in the broader population about the realities of end-of-life care and the likelihood of becoming a caregiver for a relative at the end of life. In addition, as caregivers gain experience caring for others, targeted interventions could leverage their skills and encourage them to think of engaging in end-of-life planning for themselves. The study thus offers valuable insights into the association between caregiving and end-of-life health literacy, suggesting pathways for enhancing end-of-life care. Finally, the research underscores the necessity for future studies to delve deeper into the dynamics of this relationship between providing end-of-life care to relatives and end-of-life health literacy.

## Electronic supplementary material

Below is the link to the electronic supplementary material.

Supplementary Material 1

## Data Availability

This paper uses data from Börsch-Supan, A. (2022). Survey of Health, Ageing and Retirement in Europe (SHARE) Wave 8. Release version: 1.0.0. SHARE-ERIC. Data set. 10.6103/SHARE.w8.100. Study data already de-identified are available to the scientific community upon submitting a data requestion application to the SHARE study.
