# Supplementary material for: Learning from experience: does providing end-of-life care support for relatives boost personal end-of-life health literacy?
Source: BMC Palliat Care. 2025 Jan 9;24:6. doi: 10.1186/s12904-025-01645-1 (PMC11716501; doi:10.1186/s12904-025-01645-1)
Supplement: Supplementary file 1 — Supplementary Material 1 [file 12904_2025_1645_MOESM1_ESM.docx]

**Learning from experience: Does caregiving for loved ones boost personal end-of-life health literacy?**

**Appendices**

| **Appendix 1: the 18 items from the S-EOL-HLS scale:** |
| --- |
| **Question 1:** The medical community uses specialized vocabulary. We would like to know whether it is easy or difficult for you to understand what the following medical terms means:  Answer categories: "Very easy", "Fairly easy", "Fairly difficult", "Very difficult"   1. Prognosis 2. Intubation 3. palliative care 4. cardiopulmonary resuscitation 5. artificial nutrition 6. sedation   **Question 2:** We would like to know how comfortable you feel with the following situations. For you, is it easy or difficult to…  Answer categories: "Very easy", "Fairly easy", "Fairly difficult", "Very difficult"   1. Define what is overtreatment for you? 2. Talk about your end-of-life preferences with someone you trust such as a close family member or friend? 3. Talk to a physician or other medical expert to learn more about advance care planning tools and end-of-life treatments? 4. Find information and/or obtain template forms to complete a so-called “advance directives”? 5. Make decisions on whether to accept a treatment or not based on probabilities regarding chances of different treatment outcomes? 6. Choose between comfort care (relieving suffering without slowing the disease) and aggressive life-prolonging treatment (heavy chemotherapy, intensive care with artificial ventilation) should you suffer from a terminal disease? 7. Define specific conditions or situations in which you would prefer to be left to die?   **Question 3:** Imagine being asked today to write down whether or not you would like to receive certain medical treatments in a situation in which you are no longer able to decide for yourself (advance directives). How easy or difficult is it for you to indicate today in writing whether you wish to receive or refuse the following treatments at the end of life?  Answer categories: "Very easy", "Fairly easy", "Fairly difficult", "Very difficult"   1. breathing machines 2. artificial nutrition 3. blood transfusions 4. antibiotics 5. cardiopulmonary resuscitation |

| **Appendix 2: experiences with end-of-life care support** |
| --- |
| Sometimes personal experiences influence our decisions.  **Question 1:** Have you ever made medical decisions for a person at the end of life who was close to you and who was no longer able to decide for him/herself?  Answer categories: “Yes” or “No”.  **Question 2:** Have you ever accompanied (being present, visiting, moral support) a relative or close friend at the end of life?  Answer categories: “Yes” or “No”.  **Question 3:** Have you ever cared (personal care, giving medicine, feeding) for a relative or close friend at the end of life?  Answer categories: “Yes” or “No”. |
